# Imperfections in the Cumberland Infirmary

**Published:** 1914-10-10

**Authors:** 


### Imperfections in the Cumberland Infirmary.

*To the Editor of* The Hospital.

Sir.—In The Hospital for September 19 may be found a contribution by Sir Henry Burdett, K.C.B., K.C.V.O., on which a few comments are needed. The governors, the committee, and the staff of the Cumberland Infirmary, both honorary and resident, will all feel grateful to the writer of the article for his criticisms and suggestions, even if certain inaccuracies mar the trustworthiness of his statements.

Sir Henry Burdett contrasts the condition of the infirmary in 1893 with his impressions gathered in June 1914, and it would seem that, so far from improving, the hospital has deteriorated during this period. It is true that he draws attention to certain structural improvements and additions which have cost some £30,000, but he describes a completely equipped ophthalmic department as a "new ophthalmoscope room."

I may mention one of the advances made in the Cumberland Infirmary since 1893 which Sir Henry Burdett has seemingly overlooked. In 1899 a new aseptic operating-room—one of the first in the provinces—was completed, but the Report credits us with the possession of a new clinical room to each ward, which up to the present we have not acquired.

Sir Henry Burdett causes a smile to spread when he writes: "We gathered the impression that at present there is little or no life in the place." "There was a lack of smart, contented briskness in the general atmosphere and work going on in the wards." Well, there is life and spirit enough left to repudiate so hasty a judgment, which to those who have other than a superficial knowledge of the infirmary must seem both featureless and uninformed.

Sir Henry Burdett is of opinion that "the Cumberland Infirmary is one of those institutions which require someone in authority with technical knowledge and experience, who is constantly devoting attention and time to the supervision and enforcement of smartness throughout the wards and those portions of the establishment which are included in the hospital section." One wonders from what source Sir Henry Burdett gathered that there is a pressing necessity for a sort of supervising inspector who would create the smartness which is lacking in our hospital. All of us have some Utopian plans and ideas, but it is usual to cut the coat according to the cloth in hand, and I question if a superintendent (such as may be found in larger hospitals) would help us very much, even if his services were gratuitous. Sir Henry Burdett thinks otherwise, but, in spite of the opinion his wide experiences have helped to form, I think we know how to adapt our expenditure to our means rather more certainly than he at his visit appears to have been able to do.

"The administration has been sadly changed for the worse." I pick out this condemnatory opinion with considerable amazement, to use a mild expression, and assume that the committee, being the body responsible, has to bear the brunt of this unjustifiable attack. And I am more than at a loss to see where Sir Henry Burdett can have gathered materials for so sweeping a diatribe.

I quite agree with Sir Henry Burdett "that the real happiness and efficiency of a hospital must depend largely upon the presence or absence of devotion and zeal coupled with a keen interest in the work among all sections of the staff." Sir Henry Burdett, with marvellous insight, in the course of a short visit, found the Cumberland Infirmary lacking in these attributes.

Sir Henry Burdett, in his mercy, refrains from stating the conclusions which his inquiries led him to arrive at, but this restraint is cast off in his summing-up of the committee of management, which he thinks has greatly deteriorated. It is well known that the composition of a committee depends on mixed circumstances, and our committee, in accordance with modern requirements, consists of a fair representation of all sections of the community. Sir Henry Burdett would imply that wider representation begets narrower views, for he asks: "Has there been a gradual elimination of the former principles which led some of the most distinguished residents within the county to take an active part in the management?" Sir Henry Burdett opines that only an investigation conducted by an expert could cope with what he considers to be the mismanagement of the infirmary by incapable officials, who have, in his opinion, usurped the position of control they are not properly qualified to exert. It seems that this view must be the result of some misapprehension on Sir Henry Burdett's part, for I am almost certain that the committee would repudiate the statement that there has been any interference with their scheme of management.

Then come the following recommendations: "Office work and all connected with it" must be kept "wholly separate from the internal management of this hospital." And he returns to his resident medical officer plan, and gives our secretary what is called in Cumberland "sneck possit," or the cold shoulder. The suggestion of a landscape gardener is quite a good one, but we cannot run the risk of the criticism of our subscribers even if we enjoy that of Sir Henry Burdett, but in point of fact there is not a hospital in the country with so fine a site and so splendid a distant view. We cannot afford a gardener of any kind, and there are not many living in Cumberland who keep a landscape gardener or even consult one. Nevertheless, it is a provision to be kept in mind when our affairs are sufficiently prosperous and our supporters are urging us on to take such a step.

With regard to Sir Henry Burdett's suggestion that we should have the whole of the nursing and domestic affairs directly under the control of a lady superintendent, who should have the assistance of a housekeeper, one wonders what he found so far wrong with the matron, Miss Sylvia Parker, who gave him the pleasure of taking him round the hospital. In a larger hospital the sisters, nurses, and probationers can be quite well placed all together under a superintendent of nurses, and a housekeeper employed to look after the commissariat, but in a small provincial hospital like the Cumberland Infirmary care has to be taken not to swallow up the income with a larger staff than is actually needful. Sir Henry Burdett would allow us a resident medical officer (I presume a secretary also), a lady superintendent, and a housekeeper, and these together with our two house-surgeons would make a big hole in £1,000 a year, a luxury we are quite unable to afford even if it was really essential, which I think is very doubtful.

I cannot help thinking that Sir Henry Burdett's conclusions are based either on inaccurate deductions or that he has been supplied with data that are for the most part fancies. Open as we are to suggestions, and grateful for any just criticism, I can hardly, in justice to the Cumberland Infirmary, allow some of Sir Henry Burdett's to pass without these few comments, which I have sent to the *Lancet* as well as to The Hospital.—I am, Sir, yours, etc., H. A. Lediard,

Carlisle, October 4, 1914.

